# Femtosecond Laser Trimming with Simultaneous Nanostructuring to Fine Piercing Punch to Electrical Amorphous Steel Sheets

**DOI:** 10.3390/mi12050568

**Published:** 2021-05-17

**Authors:** Tatsuhiko Aizawa, Tomomi Shiratori, Yoshihiro Kira, Tomoaki Yoshino, Yohei Suzuki

**Affiliations:** 1 Surface Engineering Design Laboratory, Shibaura Institute of Technology, Tokyo 144-0045, Japan; taizawa@sic.shibaura-it.ac.jp; 2 Faculty of Engineering, Graduate School of Science and Engineering, University of Toyama, Toyama 930-8555, Japan; 3 Graduate School of Engineering, University of Toyama, Toyama 930-8555, Japan; m1971210@ems.u-toyama.ac.jp; 4 Komatsu-Seiki Kosakusho, Co., Ltd., Suwa-City 392-0012, Japan; yoshino@komatsuseiki.co.jp (T.Y.); y-suzuki@komatsuseiki.co.jp (Y.S.)

**Keywords:** fine piercing, electrical amorphous steel sheets, induced damages by piercing, diamond coated punch, edge width, edge profile, nanostructure, iron loss

## Abstract

A CVD (Chemical Vapor Deposition) diamond coated tungsten carbide (WC) and cobalt (Co) sintered alloy punch was trimmed by the femtosecond laser machining to sharpen its edge with about 2 μm and to simultaneously make nanostructuring to its side surface. In addition to the sharpened edge, its edge profile was formed to be homogeneous enough to reduce the damage layer width by piercing the electrical amorphous steel sheet stack. Each brittle sheet in the stacked work was damaged to have three kinds of defects by piercing; e.g., the droop-like cracking in the thickness and at the vicinity of hole, the wrinkling in peak-to-valley with partial cracking on the peaks, and the circumferential cracking. When using the WC (Co) punch with the inhomogeneous edge profile in the sharpened edge width, these three damages were induced into each sheet and the maximum damage width exceeded 80 μm. When using the punch with the sharpened edge and homogeneous edge profile, the wrinkling mode was saved and the total affected layer width was significantly reduced to less than 20 μm. Through the precise embossing experiments, this effect of punch edge profile condition to the induced damages was discussed with a statement on the nanostructuring effect on the reduction of damaged width in electrical amorphous steel sheets. The developed tool with the sharpened edge and homogenous edge condition contributes to the realization of a low iron loss motor with a reduced affected layer width.

## 1. Introduction

The recent high-trend toward electric cars requires a high-capacity motor core with a low iron loss [1]. An electrical amorphous steel sheet with high strength is highlighted as a member of these motor cores [2]. Given the electrical amorphous steel sheet has a strength higher than 2 GPa and brittleness, its fine piercing process has many difficulties in net-shaping into a motor-core sheet [3,4]. During the indentation of cylindrical punch into the brittle metallic sheet, many defects are induced into the work at the vicinity of the pierced holes even without visible flaws in the macroscopic. These microscopic defects and damages can deteriorate the quality of magnetic properties and increase the iron loss in total [4]. When punching out these brittle sheets using the normally ground WC (Co) tools, many cracks are induced in the circumferential direction of the sheets. The piercing process using an electrical amorphous steel sheet has been attempted by stacking five sheets. However, the occurrence of a process-affected layer width has not been suppressed [5]. Concerning the perforation through the brittle solids, various impact perforation methods were reported in the literature [6,7,8]. These papers discussed the effect of impact loading conditions and penetrator media on the macroscopic response of glasses and amorphous metals. Little precise analyses are available regarding the microscopic mechanical response of brittle materials during the piercing process. As recently discussed on the fracture of amorphous solids [9,10], these microscopic damage processes must be described not only to reduce the damage width and cracking density but also to understand the effect of the damaging process on the physical properties of pierced amorphous sheets. The piercing process of these brittle sheets needs a new special tooling concept to minimize or reduce these defects.

Instead of the normal ground WC (Co) special tools for piercing the electrical steel sheets, the diamond-coated WC (Co) punch was employed together with the hardened core-die by low-temperature plasma nitriding [11,12]. Even under the nearly zero clearance, a higher burnished surface area ratio greater than 85% was attained up to 1000 shots in piercing. The full burnished surface area ratio could not be achieved by a bare diamond-coated WC (Co) punch. An AISI316L steel sheet was pierced with a full burnished surface area ratio. In addition, the mirror-polished and nano-textured holes were punched out to improve the quality of the pierced products. This piercing behavior of ductile sheets suggested that the trimmed diamond-coated WC (Co) punch must be preferable even to punching out of the brittle electrical amorphous steel sheets due to its edge sharpness together with its nanostructured side surface.

Among the treatment methods of diamond coating using beam irradiation processes, the femtosecond laser machining system was developed to adjust the punch head and side surface conditions, to sharpen the punch edge and to form the nanotextures onto the side surface [13,14,15]. As stated in [16,17], the treated surfaces are distorted and severely roughed by the thermal effect when using the nanosecond and continuum wave lasers. Even in case of the picosecond laser treatment, the irradiated surface is cut in by its abrasion process but no interaction occurs between the incident and scattering laser beams on the irradiated surfaces to form nanotextures.

In the present paper, a diamond coating of WC (Co) punch is treated to have a sharp edge with a width of 2 μm, as well as a nano-textured side surface with a period of 300 nm. A normally ground WC (Co) punch was prepared as a reference for the sharpened edge with a width of about 2 μm. A stack of five electrical amorphous steel sheets is employed as a work for piercing and embossing experiments. Three types of damages are induced into each sheet in the stacked work by piercing with the use of normal punch; e.g., the droop-like damage around the pierced holes, the radially wrinkling damage with peaks and valleys, and the circumferential cracking. The total damaged width becomes more than 80 μm and three times larger than the sheet thickness. When using the laser-trimmed diamond-coated punch, the wrinkling damages disappeared in all the pierced sheets of work and the total damage width in maximum was less than 20 μm. This difference in damages comes from the homogeneity in the sharp edge profile. In addition, the droop-like damage width, as well as the circumferentially cracking damage width, becomes smaller than when using the normally ground WC (Co) punch. This is because of the incremental shearing by the nanostructured side surfaces of laser-trimmed diamond-coated punch. The above reduction of induced damages by the laser-trimmed diamond-coated punch is analyzed using the precise embossing experiments to explain the mechanism to minimize the induced damages to brittle sheets by piercing.

The tooling design on the piercing punch to electrical amorphous steel sheets has a significant contribution not only to the experimental description of microscopic damages induced by piercing but also to the demonstration of the controllability of the microscopic damage process by tooling toward zero-damage induction to amorphous sheets. These tool designs reduce the microscopic damage during the shearing of electrical amorphous steel sheets and contribute to the realization of motors with reduced iron loss.

## 2. Experimental Procedure

The femtosecond laser trimming system was employed to reduce the maximum surface roughness of the diamond-coated WC (Co) punch head and side surfaces, to sharpen its edge, and to form the nanotextured side surface. This punch was inserted into a die set for a precise CNC (Computer Numerical Control) stamping system to pierce the worksheet. The effect of the edge profile and nanotextures on the piercing behavior was investigated by precise analysis on the pierced sheets.

### 2.1. Femtosecond Laser Trimming System

A femtosecond laser machining system (FEM-4; LPS-Works, Co., Ltd., Tokyo, Japan) was employed for the trimming process. Its features are listed in Table 1. The average power was 8.2 W, the maximum pulse energy was 50 μJ and the single-shot power was estimated to be 0.25 GW. The fluence was also constant by 0.6 J/cm^2^ for nanotexturing of the side surface. This fluence was reduced to 0.265 J/cm^2^ for geometric adjustment of the rough punch head.

In this femtosecond laser trimming, the punch edge is sharpened as an intersection between the trimmed punch head and side surfaces. Due to the optical interaction between the scattering beams on the rough surface and the incident beam, the laser-induced periodic surface structuring (LIPSS) takes place together with the trimming processes [14,15]. Nanotexturing to diamond films by LIPSS is made by using the nanoablation via the femtosecond laser irradiation [13,14,15]. Under the laser irradiation conditions in the above, the LIPSS period is estimated to be 250 nm [17]. The LIPSS period is dependent on the fluence. Since the fluence is 0.265 J/cm^2^, it is estimated to be 0.5 of wavelength or 250 nm after [17,18].

### 2.2. Precise CNC Stamping and Die Set

A precise CNC stamping system was employed for piercing experiments under the narrow clearance [19]. The load cell was embedded into a lower cassette die to allow in-process monitoring of the load to stroke relationship. The nanometric X-Y stage was also utilized for regular setting-up to place the punch location. Table 2 summarizes the size and dimension of punch and die together with the piercing condition. The punch and die diameter were measured by an ultrahigh accurate 3-D profilometer (UA3P-L; Panasonic Production Engineering Co., Ltd., Osaka, Japan). The punch edge radius was measured by using laser scanning confocal microscopes (KEYENCE Co., Ltd., VK-X3000, Tokyo, Japan).

### 2.3. Work Materials

**Figure 1:**
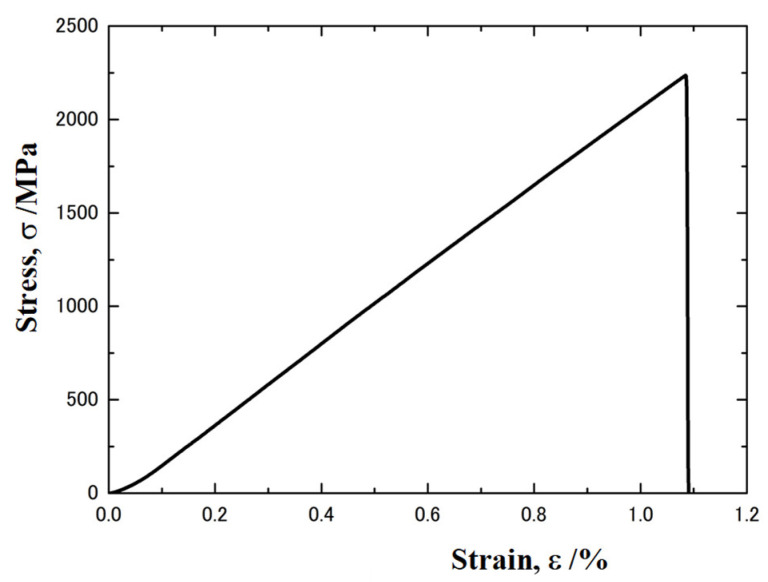
Stress-strain relationship of electrical amorphous steel sheets.

An electrical amorphous steel sheet has high strength with brittleness. Its stress-strain curve is shown in Figure 1. The ultimate strength reaches 2240 MPa at the nominal strain of 1.09%. This stress-strain curve is the result of actual measurement using a 1/2 model of JIS No. 13B tensile test piece and test speed was selected at 5 mm/min This linear relationship reveals that no plastic straining takes place in the piercing process. The thickness of the sheet was 25 μm. In the piercing experiments, a stack of five sheets was prepared and employed as a work.

### 2.4. Measurement and Observation

SEM (Scanning Electron Microscopy; JEOL Ltd., JSM-6060-EDS, Tokyo, Japan) was used to describe the microstructural change in work, as well as the punch surface conditions during the piercing process. A three-dimensional profilometer (Bruker AXS GmbH, NT91001, Karlsruhe, Germany) was also employed to describe the deformation of each constituent sheet in work before and after the punching out. An image dimension measurement system (KEYENCE Co., Ltd., IM-6000, Tokyo, Japan) was used to measure the inner diameter of the pierced hole. A load cell (Kyowa Electronic Instruments Co., Ltd., LMB-A-2KN, Tokyo, Japan) and high-accuracy CCD laser displacement sensor (KEYENCE Co., Ltd., LKG-30, Tokyo, Japan) were used to measure the piercing load and stroke, respectively. The measured piercing load and strokes were stored in a stand-alone measurement unit (KEYENCE Co. Ltd., NR-600, Tokyo, Japan).

## 3. Experimental Results

Two punches were utilized for fine piercing and embossing experiments; e.g., a WC (Co) punch with the sharped edge by grinding and a laser-trimmed diamond-coated WC (Co) punch with the sharp edge and nanotextures.

### 3.1. Punch Edge Sharpening with Nanotexturing

**Figure 2:**
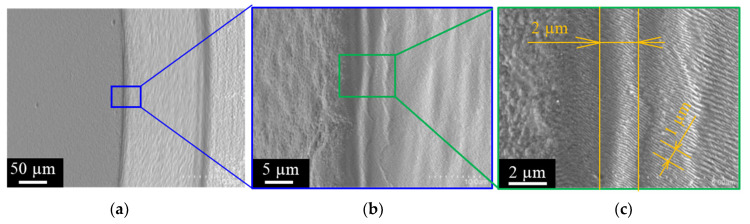
SEM image on the punch head and side surfaces of trimmed diamond-coated punch across the punch edge with various magnifications. (**a**) Overall view of the trimmed, diamond-coated punch, (**b**) Higher magnification image on the punch edge, and (**c**) Highest magnification image on the vicinity of the edge.

The diamond-coated WC (Co) punch with a diameter of 1.958 mm was prepared and trimmed by the femtosecond laser system. The same trimming conditions as defined in [15] were utilized in this processing. The diamond-coated WC (Co) punch edge radius was measured to be 2.75 μm. Figure 2 depicts the punch head, the punch edge, and the trimmed side surface. This figure was taken with the punch tilted 30 degrees from the horizontal. As shown in Figure 2a,b with lower magnification, the edge profile looks homogeneous as an intersection of trimmed head and side surfaces. Figure 2c depicts the high magnification SEM image in the vicinity of the punch edge. On the side surface of the punch, there are three grooves of about 300 nm in a width of 1 µm. The skewed nanotextures with the LIPSS-period of 300 nm were cut in with the regular alignment on the side surface just from the punch edge. Since the head portion and the side surface portion are formed under different laser conditions, a nanotexture is generated only on the side surface portion. As had been discussed in [15,18], this skew LIPSS comes from the femtosecond laser irradiation conditions on the rough diamond coating surface. The punch edge has a homogeneous shape in the range shown by the width of 2 µm.

### 3.2. Piercing Process by Edge-Sharpened WC (Co) Punch

**Figure 3:**
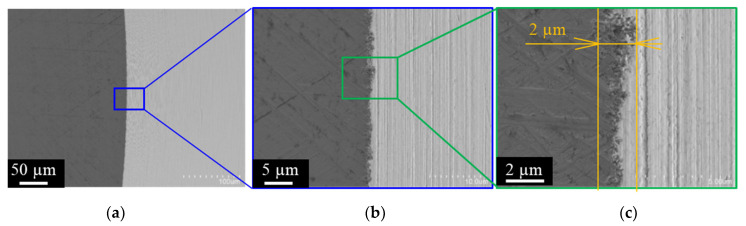
SEM image on the punch head and side surfaces of normally ground WC (Co) punch across the punch edge with various magnification. (**a**) Overall view of the trimmed, diamond-coated punch, (**b**) Higher magnification image on the punch edge, and (**c**) Highest magnification image on the vicinity of the edge.

To describe the piercing behavior of brittle work, a normally ground WC (Co) punch with a diameter of 1.986 mm was prepared and edge-sharpened to be less than 2 μm by the grinding machine. Figure 3a depicts the overall view of normally ground WC (Co) punch. Since both head and side surfaces were ground by a grinding machine, a sharp edge was formed. However, as seen in Figure 3b, the edge profile is not homogeneous but diffusing in the circumferential direction. From Figure 3c, when the edge of the punch was observed with a width of 2 µm, some WC (Co) grains were removed by grinding, and the ground punch edge was in a heterogeneous state from 1 µm to 2 µm. In the following piercing experiment, this difference in the edge profile between two punches or between Figure 2 and Figure 3 is considered as an affecting factor to the piercing process.

**Figure 4:**
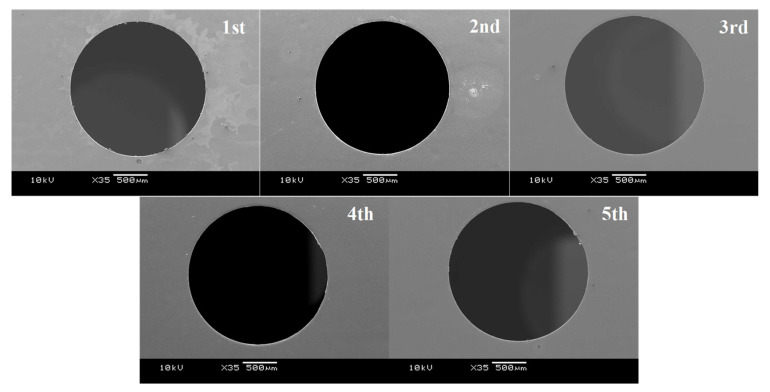
Macroscopic piercing behavior to punch out five electrical amorphous steel sheets in a stacked work by a single shot.

Work with a stack of five electrical amorphous steel sheets was pierced in a single shot to investigate the microstructure change with the use of normally ground WC (Co) punch. The punch first touched the 1st sheet and then punched five sheets up to the 5th sheet. Figure 4 depicts the macroscopic image of pierced sheets. No visible flaws are seen in each sheet.

**Figure 5:**
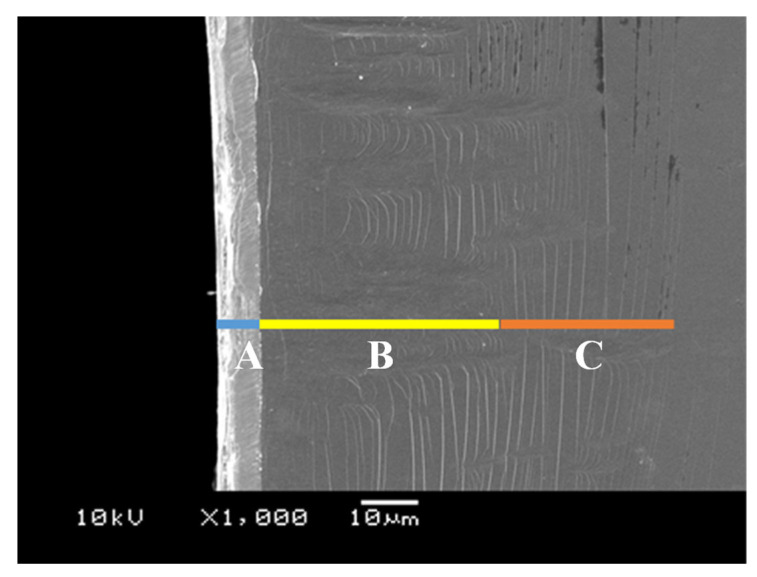
Microstructure damages of electrical amorphous steel sheet induced by piercing in a single shot with the use of a normally ground WC (Co) punch. A-defect width (W_A_) is 10 μm, B-defect width (W_B_) is 45 μm, and C-defect width (W_C_) is 30 μm, respectively.

Among five sheets, the second pierced sheet was employed to describe the induced microstructure defects into the electrical amorphous steel sheet by piercing. Figure 5 shows the SEM image at the vicinity of pierced hole edge of the sheet. Three kinds of defects were induced into a sheet by this piercing; e.g., “A” denotes a droop-like defect, “B” is a periodic defect in the radial direction, and “C” is a circumferential cracking in alignment.

**Figure 6:**
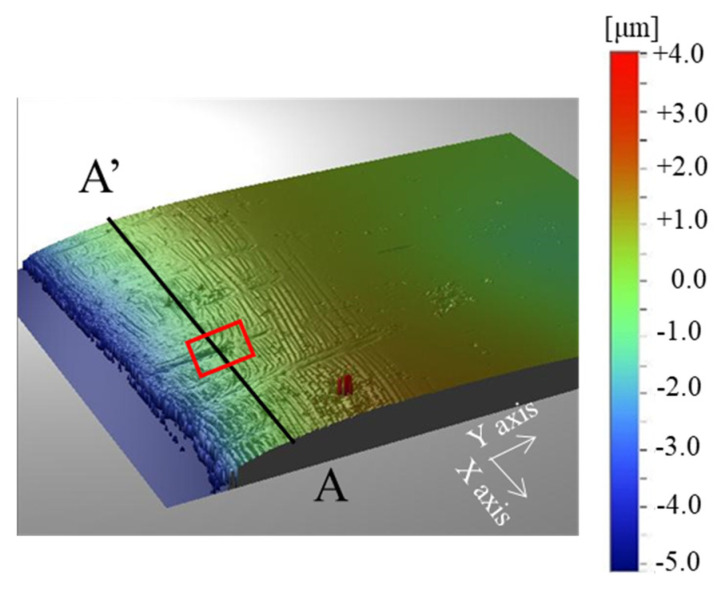
Three-dimensional shape measurement data of electrical amorphous steel sheet induced by piercing in a single shot with the use of normally ground WC (Co) punch that showed in Figure 5.

**Figure 7:**
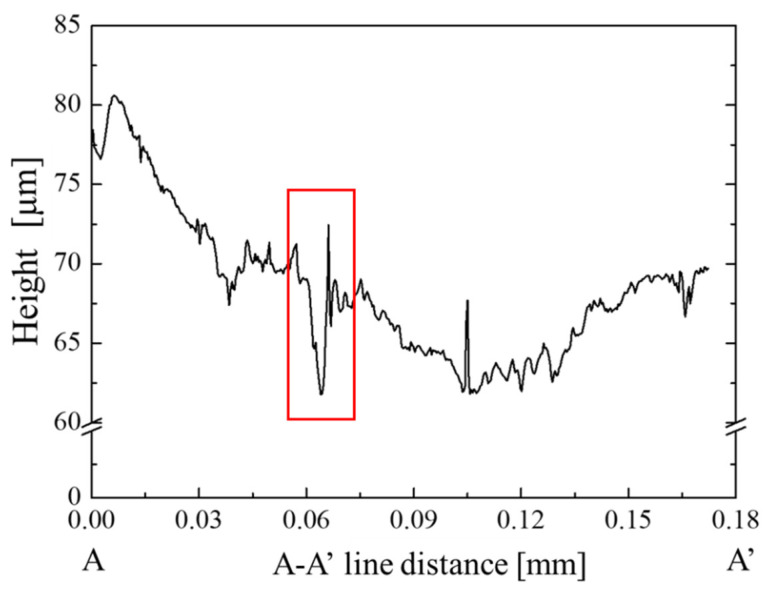
Cross-sectional shape of the “B” part of Figure 5 with the lines A-A′ in Figure 6.

Figure 6 shows the three-dimensional shape measurement data that is shown in Figure 5. The measurement range was 0.18 mm in the circumferential direction (X-axis) and 0.25 mm in the radial direction (Y-axis). From this figure, the measured surface shape is gently mountainous from the end of the hole in the circumferential direction, and cracks are generated on the surface. Figure 7 shows the result of evaluating the cross-sectional shape of the “B” part of Figure 5 with the lines A-A′ in Figure 6. From this figure, there is a wrinkle with a height of 10 µm surrounded in red at 0.063 mm on the A-A′ line. From Figure 5, Figure 6 and Figure 7, the brittle sheet cracks circumferentially in the thickness of the sheet and at the vicinity of the pierced hole to form the A-type defect. Due to no ductile deformation, these cracks are induced by the indentation of the punch edge from the sheet surface into its depth. The B-type defect where the compressed sheet surface wrinkles with the periodic peak-to-valley structure in the circumferential direction. The circumferential cracks are noticed on the peaks of this defect. The C-type depicts an alignment of circumferential cracks. This multi-cracking behavior proves that each sheet in the stack is tensioned by piercing the punch to induce these circumferential cracking in series. After the piercing process, how three defects are formed in series is not easy to analyze from SEM images in Figure 5. A new experiment is necessary to explain the failure procedure to generate these damages before punch-out the sheet.

These microscopic defects in the electrical amorphous steel sheets work as a gap conductance to increase the iron loss when using these sheets into a motor-core. How to reduce the damaged zone width by these defects in the sheet and how to be free from these defects in piercing are important for special tooling design.

### 3.3. Piercing Process by Edge-Sharpened and Nanotextured Punch

**Figure 8:**
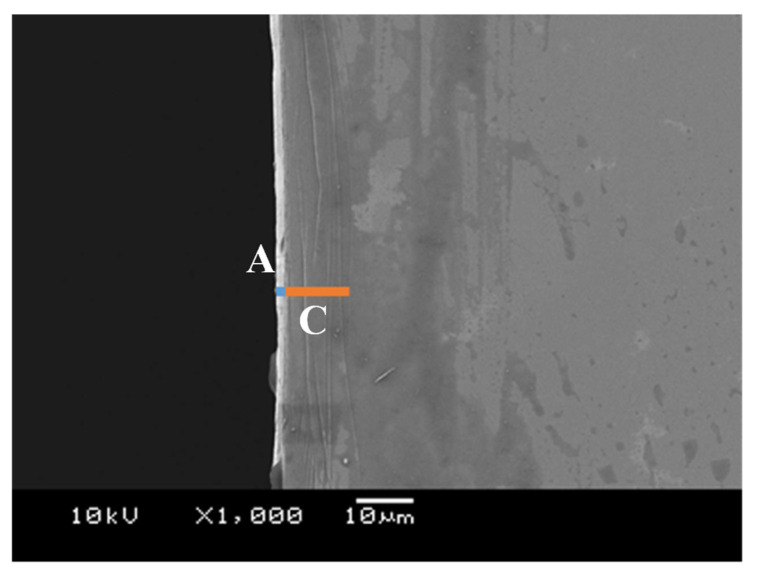
Microstructure damages of electrical amorphous steel sheet induced by piercing in a single shot with the use of the diamond-coated punch.

The diamond-coated punch with the sharp edge and nanotextured side surface was employed to pierce the electrical amorphous steel sheet similar to 3.2. Figure 8 depicts the SEM image of the second pierced sheet of work. W_A_ and W_C_ are reduced and no W_B_ is detected in comparison to Figure 5; e.g., W_A_ reduced from 10 μm to 2 μm, W_B_ reduced from 40 μm to 0 μm, W_C_ reduced from 30 μm to 10 μm, respectively.

**Figure 9:**
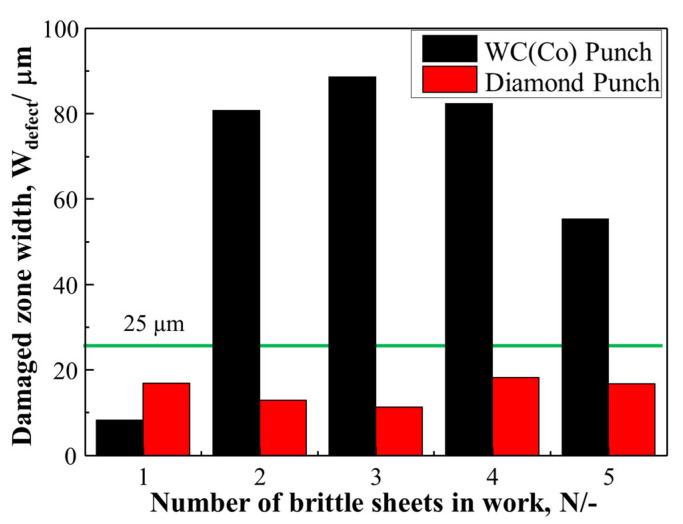
Comparison of the damaged zone width in five pierced amorphous steel sheets of work between two punches.

Figure 9 shows the comparison of the damaged zone width in five pierced electrical amorphous steel sheets of work between two punches. At the number 1 sheet, even when using the normally ground WC (Co) punch (WC (Co) punch), W_defect_ = W_A_ + W_B_ + W_C_ = 9 μm, lower than that W_defect_ = 18 μm in using the laser-trimmed diamond-coated WC (Co) punch with the sharp edge and nanotextures (diamond punch).

At the number 2 sheet, three defects were generated in the pierced brittle sheets by using the WC (Co) punch even with inhomogeneous sharp edge profile; e.g., the measured damaged zone width is W_defect_ = 80 μm as shown in Figure 5. When using the punch with an inhomogeneous sharp edge profile, the work surface is radially compressed in part so that the compressed area in part forms a peak and the un-compressed area is left as a valley. On the other hand, when using the diamond punch with the sharp edge and nano-structured side surface, W_defect_ = 12 μm, as shown in Figure 8. This disappearance of wrinkling damages by W_B_ = 0 μm comes from a uniform indention of the sharp edge. The whole work surface is uniformly compressed when using a punch with the homogeneous sharp edge profile. From the number 2 sheet to the number 5 sheet, W_defect_ by the WC (Co) punch becomes much more than W_defect_ by the diamond punch. This reveals that both the radial and circumferential defects (or B and C) are formed in the broader width by three times the sheet thickness.

### 3.4. Comparison of Piercing Behavior by Two Punches in the Formation of Affected Zones

**Figure 10:**
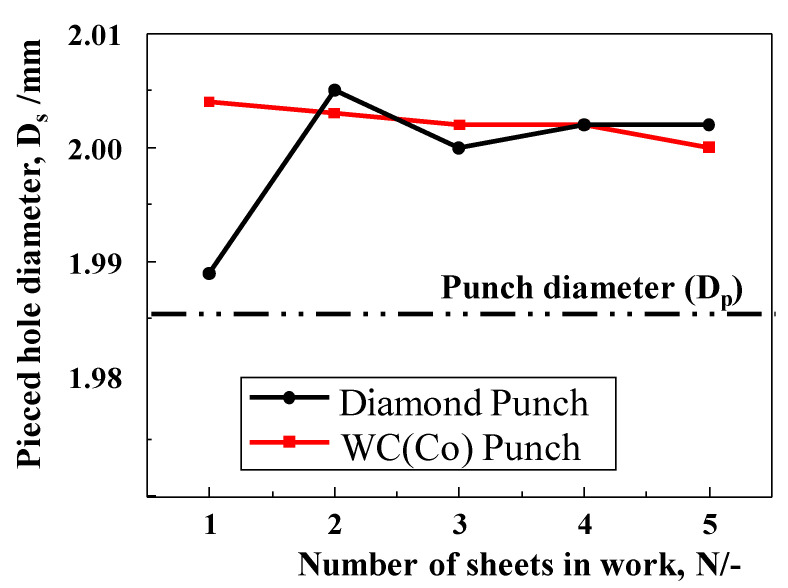
Deviation of the pierced hole inner-diameter among five electrical amorphous steel sheets when using two punches.

Figure 10 shows the inner diameter of the pierced hole among five electrical amorphous steel sheets when using two punches. D = 2.003 mm is the average of the pierced hole diameter by using the WC (Co) punch; D > Dp by 20 μm, irrespective of the number of sheets. On the other hand, when using the diamond punch, D = 1.989 mm, for the 1st sheet; D > Dp only by 4 μm. From sheet number 2 to 5; no difference was seen in D between piercing processes by two punches.

**Figure 11:**
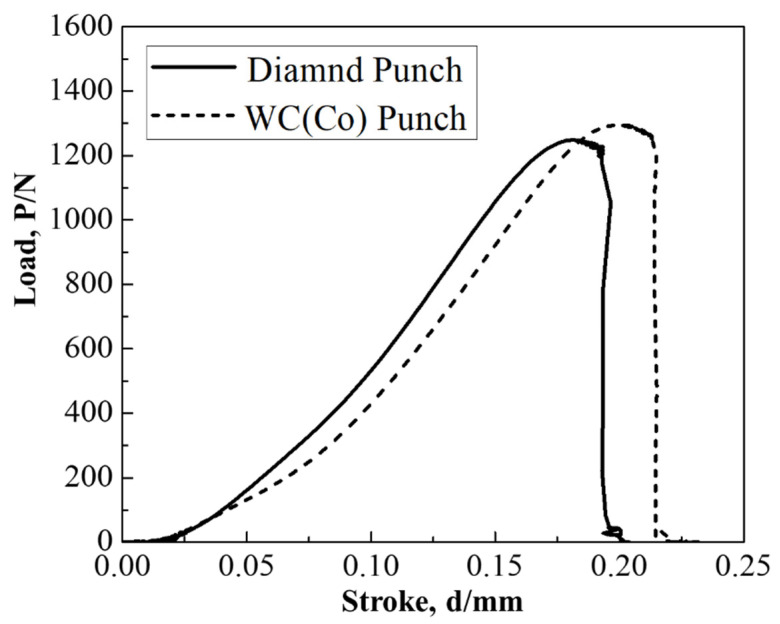
Comparison of the load–stroke relationships between the piercing processes by two punches.

The load–stroke relationships are compared in Figure 11 to investigate the difference in mechanical response when piercing with the use of the WC (Co) punch and diamond punch. When using the WC (Co) punch, the maximum load (P_MAX_) was 1.3 kN and the maximum stroke (δ_MAX_) was 0.23 mm. When using the diamond punch, P_MAX_ was 1.25 kN and δ_MAX_ was 0.20 mm. This reduction of P_MAX_ and δ_MAX_ comes from the decrease of applied energy by the stress concentration during the piercing process.

### 3.5. Piercing Process Analysis

The difference in the piercing performance between the two punches revealed that both the edge profile condition and the nanostructured side surface had a significant influence on the piercing process of the electrical amorphous steel sheet. A precise embossing experiment was employed to experimentally describe the mechanical response of an electrical amorphous steel sheet by controlling the punch indentation. In particular, the stroke (δ) was stopped at δ_failure_ − 30 μm and δ_failure_ − 10 μm, respectively, for δ_failure_ where the stacked work by five sheets was completely punched out.

**Figure 12:**
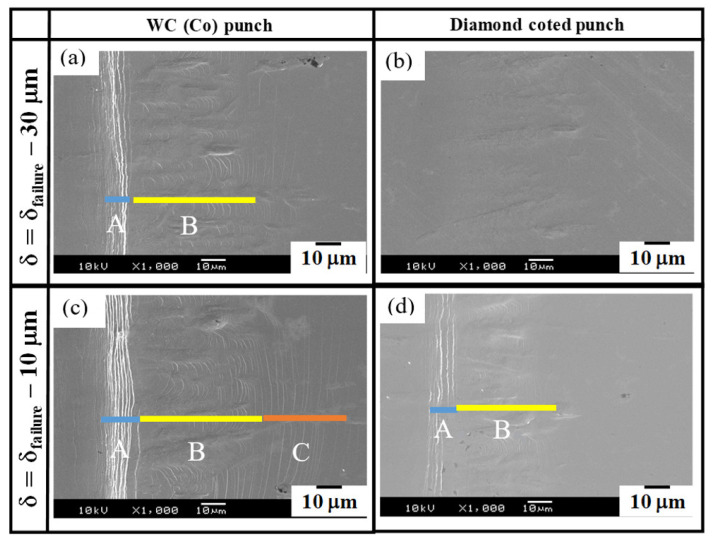
Variation of the 3rd electrical amorphous steel sheet in the stacked work by controlling the stroke δ during embossing with the use of two punches. δ_failure_ denotes the critical stroke when the work is punched out. (**a**) Using WC(Co) punch and δ = δ_failure_ − 30 μm, (**b**) Using diamond coted punch and δ = δ_failure_ − 30 μm, (**c**) Using WC(Co) punch and δ = δ_failure_ − 10 μm, (**d**) Using diamond coted punch and δ = δ_failure_ − 10 μm.

Figure 12 compares the surface damage on the third electrical amorphous steel sheet in the stacked work, induced at δ = δ_failure_ − 30 μm and δ = δ_failure_ − 10 μm, respectively, between the two punches. When using the WC (Co) punch, A-type and B-type defects were induced even at δ = δ_failure_ − 30 μm, as shown in Figure 12, and C-type defect was additionally generated at δ = δ_failure_ − 10 μm, as shown in Figure 12c. This proves that the circumferential cracks are significantly induced into the electrical amorphous sheets just before their complete punch-out. On the other hand, no defects were seen on the SEM image on the sheet surface at δ = δ_failure_ − 30 μm when using the diamond punch as shown in Figure 12b. Even at δ = δ_failure_ − 10 μm, only A-type defects, as well as weak B-type defects, were generated in the sheet as shown in Figure 12d. This weak B-type is formed by shallow wrinkles and no cracks have occurred.

**Figure 13:**
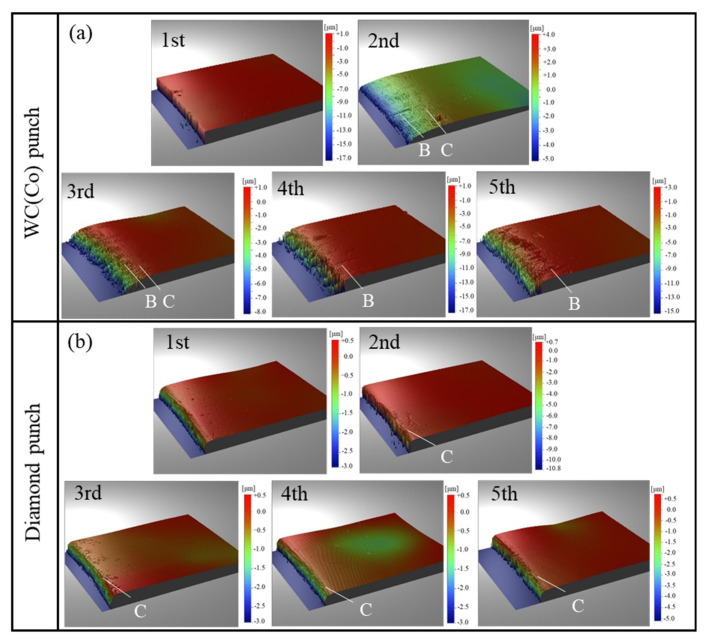
The surface profile of the pierced sheets in the radial direction from the hole. (**a**) Piercing by the WC (Co) punch, and (**b**) piercing by the diamond punch.

Figure 13 shows the surface profile of pierced sheets in the radial direction from the hole between two punches. When using the WC(Co) punch, the 2nd sheet to the 5th sheet have a B-type defect, and the 2nd and 3rd sheet have C-type defects that are shown in Figure 13a. On the other hand, when using the diamond punch, there are only seen in C-type defects from the 2nd to 5th sheet near the hole as shown in Figure 13b. The differences in the embossing behavior between two punches proved that deformation of the electrical amorphous steel sheet was suppressed by the stress concentration just below the diamond punch edge and that defects are induced by the stress redistribution in the radial and circumferential directions when embossing the electrical amorphous steel sheet by the WC (Co) punch.

## 4. Discussion

In the piercing of the ductile electrical amorphous steel sheets, a droop was first formed by the initial indentation of punch edge into the sheet and was followed by the elastoplastic straining in the shearing and bending modes as reported in References [3,5,11,12]. Through the systematic studies on the piercing behavior by varying the edge sharpness [20,21], the punch edge sharpness under the narrow clearance influences the quality of the pierced steel sheet. Owing to the ductility of sheet materials, no damages in Figure 5 were experienced in those piercing processes. In case of piercing the brittle amorphous sheets, no plastic deformation occurs but defects, as well as cracks, are generated into the pierced sheets even in macroscopic [5]. Three defects are induced in Figure 5 and the total damage width exceeds 80 μm in Figure 9 when using the WC (Co) punch with the inhomogeneous edge profile. Other geometric and topological features of the piercing punch than the edge sharpness are required for reduction of these induced damages by piercing.

Let us consider the role of shape edge profile in the piercing process. Through indentation of the punch edge to a brittle sheet, the convex parts of the inhomogeneous edge profile compress the sheet to form a peak into the sheet. The concave parts are retarded to cut into the sheet; amorphous work between the adjacent peaks turns to be a valley. Inhomogeneity in the edge profile induces wrinkling damage with the peak-to-valley distortion. Since these induced peaks are tensioned, the circumferential cracking occurs only on them. When punching the number 1 sheet with WC (Co) punch as shown in Figure 8, the damaged zone width was reduced compared to the result of punching with diamond punch, which considered that the sharp edge condition in inhomogeneous punch edge worked effectively. When using the punch with a homogeneous edge profile, no periodic peak-to-valley defects were formed by piercing the punch and the C-type cracks were only seen near the hole. As proven in Figure 8 and Figure 12, no wrinkling defects are induced when using the punch with a homogeneous edge profile. Inhomogeneity in sharpening the punch edge is responsible for this formation of wrinkling defects.

What kind of roles does the nanotextured punch side surface play? In piercing the ductile sheets, this nanotexture works as a nano-channel to eject the debris fragments out of the piercing field [14,22]. This ejection mechanism prolongs the lifetime of the piercing punch. In the case of piercing the brittle sheets, each nanostructure works as an edge to sustain the strain concentration in piercing. In Figure 12, three damages were induced when using the WC (Co) punch while no damages were seen at δ_failure_ − 30 μm when using the diamond-coated punch. In the former, the punch has one sharp edge so that the applied stress redistributes sheet-by-sheet during piercing the stacked work. On the other hand, each nanotexture works as a successive edge to preserve the stress concentration so that the damage width is reduced in each sheet in Figure 9 and the sheet surface profile becomes more homogeneous in Figure 13. Compare the formation of A- and C-type defects between Figure 5 and Figure 8; the number of circumferential cracks decreases by the nanostructured punch. This reveals that stress concentration is sustained to suppress the generation of droop-like defects in piercing.

Let us certify the order to generate the three defects into the amorphous sheets by piercing. The surface profiles are compared between two punches, in Figure 12 and Figure 13 and at the strokes of δ_failure_ − 30 μm and δ_failure_ − 10 μm, respectively. A droop-like defect is first induced at the initial indentation of punch edge in common to two punches. When using the nanostructured punch, the number of cracks is reduced and the distance between adjacent cracks is broadened by successive penetration of nanotextures into the amorphous sheet. This generation of A-type defect is followed by a B-type defect when using the punch with an inhomogeneous edge profile. No B-type defect was induced when using the punch with a homogeneous edge profile. Finally, C-type defects are generated when the stress concentration is not sustained in piercing.

## 5. Conclusions

Two punches with the same edge sharpness of 2 μm were used to pierce the electrical amorphous steel sheet stack. When using the normally ground WC (Co) punch, three kinds of defects were always formed at the vicinity of pierced holes, irrespective of the number of sheets in the work. Total damaged zone width reached 90 μm in maximum, and the inner diameter of holes expanded by 20 μm on average from the outer diameter of the punch. The surface profiles in five pierced electrical amorphous sheets have significant inhomogeneity with distributed defects. On the other hand, the maximum damaged zone width was limited by 20 μm and every pierced electrical amorphous steel sheet had homogeneous surface profiles due to the homogenous edge profile and nanostructures on the side surface of the laser-trimmed diamond-coated punch. In particular, the homogeneous edge profile is responsible to save the wrinkling defects, and the nanostructures work as successive edges to reduce the defect density, as well as damaged zone width. These reductions in defect density and damaged zone width contribute to reducing iron loss. Most functional metals and alloys with high strength have little ductility. Their normal embossing and piercing processes inevitably experience the generation of defects and damages to deteriorate their intrinsic functional properties. The femtosecond laser trimming can fabricate the complex-shaped punch and core-die with proof of their edge sharpness, homogeneity in edge profile, and periodic nanostructures in their surfaces. These piercing tools induce little defects even in microscopic to their sheet products.

## Figures and Tables

**Table 1:** Features of femtosecond laser trimming system.

Wavelength	515 nm
Pulse duration	180 fs~190 fs
Average power	8.2 W
Repetition	400 kHz
Beam spot size	φ10.5 μm ± 0.75 μm
Work piece rotation speed	120 degree/s

**Table 2:** Size and dimension of punch and die with the piecing condition.

	Tool Type	Parameter
Punch	Laser-trimmed diamond-coated	φ1.985 mm
	Ground WC(Co)	φ1.986 mm
Die	Ground WC(Co)	φ2.002 mm
Clearance	−	0.008 mm
Piercing speed	−	5 mm/s
